# The effect of bilateral rectus sheath and oblique subcostal transversus abdominis plane blocks on mechanical power in patients undergoing laparoscopic cholecystectomy surgery: a randomized controlled trial

**DOI:** 10.1186/s12871-025-03062-6

**Published:** 2025-04-16

**Authors:** Esma Karaarslan, Yasin Tire, Mahmut Sami Tutar, Nuran Akıncı, Hasan Alp Mermer, Sami Uyar, Dilek Ateş, Gürcan Şimşek, Betül Kozanhan

**Affiliations:** 1 Department of Anesthesiology and Reanimation, Konya City Hospital, Konya, Turkey; 2 General Surgery Department, Konya City Hospital, Konya, Turkey; 3 Department of Anesthesiology and Reanimation, Konya Beyhekim Training and Research Hospital, Konya, Turkey

**Keywords:** Mechanical power, Rectus sheath blocks, OSTAP blocks, Laparoscopic cholecystectomy surgery

## Abstract

**Background:**

In this study, we aimed to investigate the effects of bilateral rectus sheath blocks (RSBs) and oblique subcostal transversus abdominis plane (OSTAP) blocks on mechanical power (MP) in patients receiving laparoscopic cholecystectomy under general anesthesia. Additionally, we sought to evaluate the impact of these blocks on postoperative pain and quality of patient recovery.

**Methods:**

In this prospective, double-blind study, 66 patients who underwent laparoscopic cholecystectomy were randomized into two groups: Group C (control), which received a standard analgesic intravenous regimen; and Group B (block), which received bilateral RSB and OSTAP blocks. Intraoperative mechanical power was measured for all patients. Postoperative pain was assessed using visual analog scale (VAS) scores, and recovery quality was measured using the 15-item quality of recovery (QoR-15) questionnaire.

**Results:**

The mechanical power values for patients in Group C were consistently greater at all measured times: baseline, before bridion, and after bridion. Although the difference at baseline was not statistically significant, significant differences were observed before and after bridion (p values = 0.112, 0.021, and 0.003, respectively). Patients in Group B exhibited significantly lower VAS scores at all time points (30 min, 2 h, 8 h, and 24 h) (*p* < 0.05). Additionally, essential variations were noted in the administration of rescue analgesia between the groups (*p* < 0.001). Regarding tramadol consumption, Group C patients had significantly greater values [84 (74–156) vs. 0 (0–75), median (25–75th percentiles)] (*p* < 0.001). For the QoR-15 scores, Group C also had significantly greater values [129 (124–133) vs. 122 (115–125), median (25–75th percentiles)] (*p* < 0.001).

**Conclusions:**

Bilateral RSB and OSTAP blocks significantly reduce mechanical power during surgery. Moreover, they significantly decrease postoperative pain and analgesic consumption and increase patient recovery scores.

**Trial registration:**

The study protocol was registered in the international database ClinicalTrials.gov (registration no. NCT06202040). This study was conducted between December 2023 and January 2024 at the Department of Anesthesiology and Reanimation of Konya City Hospital.

**Supplementary Information:**

The online version contains supplementary material available at 10.1186/s12871-025-03062-6.

## Introduction

Laparoscopic cholecystectomy (LC) is the most common intraabdominal surgical procedure worldwide [1]. Despite advancements in anesthesia and surgery, postoperative pain following LC remains a significant issue [2, 3]. The objectives of perioperative pain management are to alleviate pain, facilitate early mobilization, ensure rapid discharge, and increase patient satisfaction. Traditional opioid-based pain management increases side effects such as excessive sedation, respiratory depression, postoperative nausea and vomiting (PONV), and impaired recovery [4]. Therefore, multimodal analgesia strategies are preferred to minimize opioid-related side effects while ensuring adequate pain control.

Various regional anesthesia and analgesia techniques are routinely employed under ultrasound guidance as part of multimodal analgesia to provide sufficient postoperative analgesia [5]. The oblique subcostal transversus abdominis plane (OSTAP) block, described by Hebbard in 2009, is a regional anesthesia technique used in mid- and upper-abdominal surgeries [6, 7]. Several studies have demonstrated that OSTAP block not only reduces postoperative analgesic and opioid requirements but also improves the quality of postoperative pain control [8, 9].

Schleich used the rectus sheath block (RSB) in 1899 for muscle relaxation and postoperative analgesia in abdominal surgery [10, 11]. Ultrasound-guided RSB is a simple, practical analgesic procedure ideal for midline or laparoscopic surgery with an umbilical incision [12]. RSB provides analgesic effects for 7 to 8 h. Nevertheless, Kim et al. reported that, compared with placebo, bilateral RSB only reduced superficial pain severity within the first hour after robotic cholecystectomy [13].

The concept of mechanical power (MP) in mechanical ventilation (MV) can be derived from the first law of thermodynamics, which states that energy cannot be created or destroyed. During MV, as pressure is generated to deliver air to the lungs, energy is generated through electrical, potential, kinetic, or thermal processes. This energy transfer can cause structural changes at the cellular and tissue levels, potentially leading to lung parenchymal damage [14, 15]. The energy transferred from the ventilator to the lungs with each breath is referred to as ‘mechanical energy’, and the amount of energy transferred per unit of time is referred to as the MP. Traditionally, MP is expressed in Joules per minute in respiratory physiology.

While MV is a life-supporting treatment, it can also potentially damage lung structures, a phenomenon known as ventilator-induced lung injury (VILI) [14, 16, 17, 18]. The degree of VILI has recently been associated with the amount of energy transmitted to the respiratory system by the MV within a specific time frame [19]. Since the COVID-19 pandemic, there has been an increased search for parameters that can help reduce lung damage from VILI and acute respiratory distress syndrome (ARDS). In this context, promoting the more widespread use of mechanical power is crucial for lung protection.

The primary aim of our study was to investigate the effects of bilateral RSB and OSTAP blocks on MP in patients undergoing LC. Additionally, as a secondary objective, we aimed to compare analgesic efficacy by performing visual analog scale (VAS) assessments on patients who did and did not receive the block during the postoperative period. Furthermore, the quality of patient recovery was assessed via the 15-item quality of recovery (QoR-15) test.

## Materials and methods

### Ethics approval and registration

Ethical approval was obtained from the Ethics Committee of Ankara Bilkent City Hospital (E1-23-4243; numbered December 15, 2023, December) for this prospective, randomized, controlled, and double-blind study. The study protocol was registered in the international database ClinicalTrials.gov (registration no. NCT06202040), with the study starting at 2023-12-22 and completing at 2024-01-25. We used the CONSORT checklist when writing our report.

All patients provided written informed consent prior to their participation in the study and before any procedures were conducted. The study protocol conforms to the 2013 Declaration of Helsinki ethics guidelines.

### Patient population and inclusion/exclusion criteria

This study was conducted between December 2023 and January 2024 at the Department of Anesthesiology and Reanimation of Konya City Hospital. The participants included patients aged between 18 and 65 years with an American Society of Anesthesiologists (ASA) classification of 1–2 who were undergoing elective laparoscopic cholecystectomy. We excluded patients who were hypersensitive to the drugs used in the study; had coagulation disorders, infections at the block site, mental retardation, severe chronic obstructive pulmonary disease (COPD), or uncontrolled bronchial asthma; had decompensated heart failure (New York Heart Association class 3–4); a history of previous lung surgery, chronic pain and treatment; morbid obesity [body mass index (BMI) > 35]; pregnancy; or who underwent open cholecystectomy or refused to participate in the study. Patients were randomized into two groups using sequentially numbered opaque envelopes, with the allocation process conducted by an anesthesia specialist who had no involvement in the study. This method ensured proper allocation concealment, preventing the randomization sequence from being disclosed until interventions were assigned. The same anesthesia specialist generated the random allocation sequence and performed the group assignments but played no further role in the study. Additionally, the individuals responsible for intraoperative measurements and postoperative follow-up were blinded to the patients’ group assignments, maintaining the integrity of the blinding process. The block group (Group B) consisted of patients who received general anesthesia and bilateral RSB and OSTAP blocks before surgery began. The control group (Group C) consisted of patients who received general anesthesia and intravenous analgesia approximately 30 min before the end of the operation.

### Standard general anesthesia and postoperative

General anesthesia was standardized and administered to all patients included in the study. Before the procedure, each patient underwent monitoring, including an electrocardiogram (ECG), peripheral oxygen saturation (SpO2), noninvasive blood pressure, and end-tidal carbon dioxide (ETCO2) measurements. A peripheral intravenous catheter (18–20G) was inserted, and crystalloid fluid maintenance was adjusted to 10 ml/kg/hour on the basis of the ideal body weight.

Anesthetic agents were administered on the basis of ideal body weight [IBW = height (cm) − 100 (for males) and 105 (for females)].

During anesthesia induction, midazolam at a dose of 0.01–0.02 mg/kg, fentanyl at 2 mcg/kg, propofol at 1–2 mg/kg, and rocuronium at 0.6 mg/kg were administered. Sevoflurane at a 2% concentration and remifentanil infusion at 0.1–0.2 µg/kg/min were utilized throughout anesthesia maintenance. Following tracheal intubation, the mechanical ventilation settings were standardized for all patients. The TV was adjusted to 6–8 ml/kg according to the IBW. A positive end-expiratory pressure (PEEP) of 5 cm H2O was applied to all patients, and the respiratory rate was adjusted to maintain an ETCO2 between 30 and 35 mmHg. Additionally, an orogastric tube was inserted to aspirate the gastric contents.

Patients’ initial measurements were taken immediately before any intervention after tracheal intubation and recorded as the baseline measurement (1st MP measurement). Three consecutive measurements were taken for all recorded measurements, and the average of these three measurements was recorded.

Following the initial measurement, patients in Group B underwent bilateral RSB and OSTAP block procedures for postoperative analgesia, which were performed by the same individual to standardize the procedure.

### OSTAP block procedure

During the OSTAP block, ultrasonography (USG) guidance, a linear probe, and a 100 mm 22 G needle were used to perform the block via an in-plane technique. A total volume of 20 cc was prepared, consisting of 10 cc of 0.5% bupivacaine and 10 cc of normal saline. The bilateral OSTAP block was administered at 10 cc to each side, resulting in a total volume of 20 cc for bilateral application. The linear probe was positioned parallel to the lower edge of the rib cage on the anterior abdominal wall. The junction of the external oblique, internal oblique, transversus abdominis, and rectus muscles was visualized. Under direct ultrasound guidance, the needle tip was advanced toward the target area, the TAP space between the internal oblique and transversus abdominis muscles. Once the needle tip was visualized within the TAP, the prepared medication was administered after confirming negative aspiration. Additionally, the spread of the medication at the junction of the rectus abdominis muscle and the TAP space was observed under ultrasound guidance.

### Rectus sheath block procedure

When the patient was supine, the ultrasound linear probe was held in the transverse plane where the posterior rectus sheath was best visualized, just above the umbilical level. Under ultrasound guidance, a 100 mm 22 G needle was used with an in-plane technique to pass through the rectus muscle. The prepared medication was administered between the rectus muscle and the posterior rectus sheath. A total volume of 20 cc was prepared for this block, consisting of 10 cc of 0.5% bupivacaine and 10 cc of normal saline. Bilateral RSB was performed for a total volume of 20 cc and 10 cc on each side.

After the block was applied to the patients in Group B and no interventional procedures were performed on the patients in Group C, all patients were transferred to the surgical team for the operation.

### Surgical procedure

Carbon dioxide insufflation was performed by inserting a 10 mm trocar into the abdomen through an incision under the umbilicus. Then, a second 10 mm trocar was inserted approximately 3 cm below the xiphoid process under camera vision. Then, a third 5 mm trocar was inserted 5 cm below the intersection of the right arcus costarium and the anterior axillary line. A fourth 5 mm trocar was inserted into the abdominal cavity 2 cm below the intersection of the right arcus costarium and the mid-axillary line. During the operation, intra-abdominal pressure was kept constant at 12 mmHg. The gallbladder was taken out of the abdomen through the incision under the xiphoid. A drain was inserted from the 3rd trocar site in patients who needed a drain. After the operation, only the fascia of the sub-umbilicus trocar site was repaired with sutures. Only the skin was sutured at the other trocar sites.

Approximately 30 min before the end of the surgery, tramadol 2 mg/kg and tenoxicam 20 mg were administered to patients in Group C.

At the end of the surgery, the second measurement (MP 2nd measurement) was taken before the awakening process started. Following this measurement, the anesthetic agents were discontinued, and the patients were ventilated with 100% oxygen. Sugammadex at a dose of 2–4 mg/kg was administered to antagonize the effects of rocuronium. Once the train of four (TOF) ratio was > 0.90, the third measurement (mec power 3rd measurement) was taken before the patients were extubated. After the final measurement, patients were extubated and transferred to the postanaesthesia care unit (PACU) for routine monitoring.

In the PACU, patients were monitored via standard techniques, including a noninvasive blood pressure cuff, ECG, and pulse oximetry. All patients were routinely observed for one hour postoperatively. The patient was first provided with information regarding the VAS score. A line measuring 10 cm was drawn horizontally, with zero (0) indicating no pain and ten [10] indicating the most intense pain imaginable. The patients were asked to select a point on this scale corresponding to their pain level. During this hour, the VAS score was queried and recorded at the 30th minute, and patients with a VAS score of 4 or higher were administered rescue analgesia with 1 mg/kg tramadol diluted in 100 cc of normal saline. After one hour of monitoring, patients were transferred to the ward.

Additionally, in the ward, all patients were revisited at postoperative hours 2, 8, and 24, during which VAS scores were recorded. Patients with a VAS score of 4 or higher at these time points were administered rescue analgesia. Finally, at the 24th hour, the QoR-15 questionnaire was administered and recorded [20].

During the postoperative period in the ward, patients received the following analgesia protocol, which the general surgery clinic routinely uses for patients undergoing laparoscopic cholecystectomy: intravenous paracetamol 15 mg/kg every 8 h. The intramuscular dose of diclofenac was 1.5 mg/kg every 12 h (maximum 75 mg).

### Calculation of the mechanical power

There are various methods available for measuring mechanical power. This study used a shortened and validated method explicitly developed during the pandemic for mechanical power measurement. This formula can be expressed as follows:

Mechanical power (J/min) = 0.098 × RR × Vt × [PEEP + ½(Pplat– PEEP) + (Ppeak − Pplat)] [21].

Therefore, mechanical power is calculated from breathing, which is the product of volume and pressure (tidal volume × pressure) multiplied by the respiratory rate of individual breaths.

### Formun Üstü

#### Outcome measures

Primary outcome measures were assessed via intraoperative measurements obtained from the mechanical ventilator. Mechanical power was calculated from these measurements (MP 1, MP 2, and MP 3).

Secondary outcome measures, including the VAS score and QoR15 score, were recorded. Postoperative assessments of the VAS score were conducted at 30 min, 2 h, 8 h, and 24 h after surgery. Additionally, at 24 h, the QoR-15T was used to evaluate the quality of patient recovery.

The preoperative age, weight, height, sex, ASA score, and BMI were recorded for all patients in both groups.

In this study, one individual performed nerve blocks, an anesthesiologist assessed pain via VAS scores, and a different individual evaluated patient recovery quality via the QoR-15T.

### Statistical analysis

The primary outcome of this study was the difference in the MP ratio between the groups. In our pilot study of 20 patients conducted before the research, the change in the MP ratio was − 2.69 ± 5.13 in the group that received a postoperative block and 5.57 ± 10.58 in patients who received multimodal analgesia. Using the data from our pilot study, calculations with a Cohen’s D effect size of 0.992 in an independent group t test model revealed that 28 patients per group (a total of 56 patients) needed to be included in the study to achieve 95% power and a maximum type 1 error of 5%. Considering the dropout probability, the necessary sample size was calculated as 33 patients per group (66 patients). Once the sample size was reached, the study was completed.

The IBM-Statistical Package for Social Sciences (IBM-SPSS, Inc., Chicago, IL, USA) version 22.0 was used to analyze the data obtained in the study. The normality of the distribution of the data was assessed with the Shapiro‒Wilk test. Continuous variables are expressed as the mean and standard deviation (SD) or median (25–75th percentile) depending on their distribution status, whereas categorical variables are presented as numbers and percentages. The ‘independent samples T test’ was applied to analyze continuous variables when parametric test assumptions were met; otherwise, the “Mann‒Whitney U test” was used. The chi-square test was used for the analysis of categorical variables. “Analysis of variance (ANOVA)” was used for repeated measures at different times between groups. The level of statistical significance was set at *p* < 0.05.

## Results

**Fig. 1:**
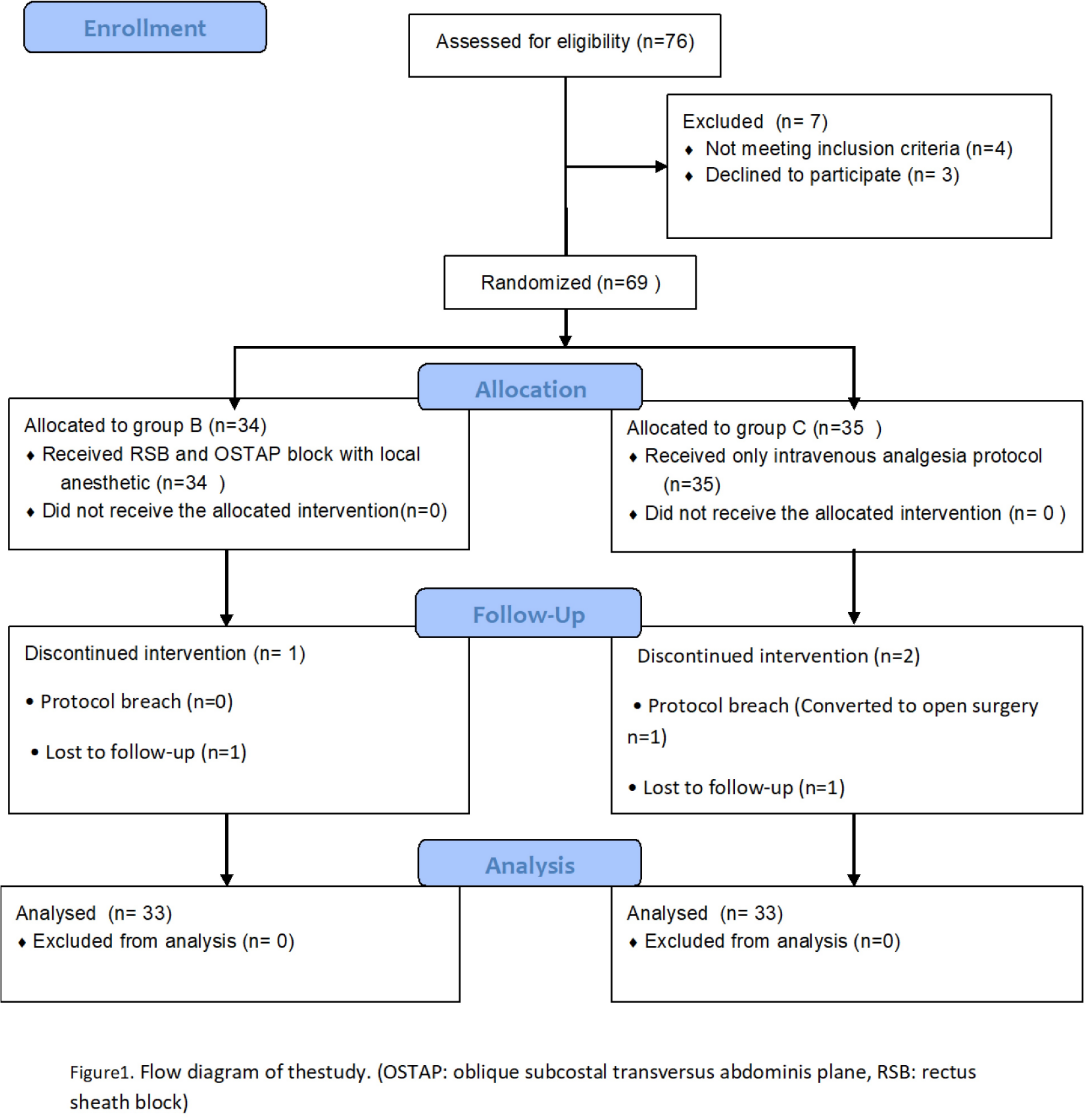
Flow diagram of the study

Seventy-six patients were first assessed for eligibility in this study; however, seven were excluded because they refused participation or met other exclusion criteria. The remaining 69 patients were allocated, randomized, and treated according to the protocol. One participant in Group B and two in Group C were excluded from the data analysis during follow-up (Group C, *n* = 33; Group B, *n* = 33). Figure 1 shows the flow diagram for patient recruitment.

When the patient characteristics of the groups were evaluated, all the parameters (weight, height, BMI, age, comorbidities, and sex) were significantly similar across the groups (*p* > 0.05) (Table 1).

**Table 1:** Patient demographic characteristics

Characteristics		Group C, *n*:33	Group B, *n*:33	*p* value
Weight (kg)		79 ± 9	75 ± 11	0,133
Height (cm)		168 ± 8	167 ± 8	0,718
BMI (kg/m2)		27,8 ± 2,05	26,67 ± 2,87	0,071
Age (year)		45 ± 11	44 ± 11	0,511
ASA n(%)	1	8(24,2%)	13(39,4%)	0,186
	2	25(75,8%)	20(60,6%)	
Additional disease, n(%)	No	18(54,5%)	22(66,7%)	0,314
	Yes	15(45,5%)	11(33,3%)	
Gender, n(%)	Man	9(27,3%)	9(27,3%)	1
	Woman	24(72,7%)	24(72,7%)	

Continuous variables are expressed as the mean ± SD, while categorical variables are shown as n(%).

When patients were assessed for mechanical power, the values for Group C were greater at all times (after “basal,” “before bridion,” and “after bridion”). Although the difference at the “basal” time was not statistically significant, “before bridion” and “after bridion” were necessary (*p* = 0.112, *p* = 0.021, and *p* = 0.003, respectively). In terms of the change from the basal time to the “before bridion” time, the percentage change in Group C was 0.40 (-0.42–2.29) (median (25–75 percentiles)), whereas in Group B, it was − 0.29 (-1.95–0.61) (median (25–75 percentiles)). This difference was statistically significant (*p* = 0.013). When looking at the change from the basal time to the “after bridion” time, the percentage change in Group C was 1.68 (0.16–4.75) (median (25–75 percentiles)), whereas in Group B, it was − 1 (-2.02–-0.18) (median (25–75 percentiles)). This difference was statistically significant (*p* < 0.001) (Table 2). When the change in the mechanical power over time was evaluated (time × group interaction), the interaction effect was significant (*p* < 0.001) (Table 2).

**Table 2:** Mechanical power values by group

Mechanical power time	Group C, *n*:33	Group B, *n*:33	*p* value
Basal	6,08 ± 0,98	5,71 ± 0,88	0,112
Beforebridion	6,17 ± 0,88	5,65 ± 0,89	0,021
After bridion	6,29 ± 0,89	5,61 ± 0,87	0,003
Mean differences percentages(“basal” to “before bridion”)	0,40(-0,42–2,29)	-0,29(-1,95–0,61)	0,013
Mean differences percentages(“basal” to “after bridion”)	1,68(0,16–4,75)	-1(-2,02–-0,18)	0,001

Variables are expressed as the mean ± SD or median (25th–75th percentiles).

When the VAS scores of patients in the groups at all time points (30 min, 2 h, 8 h, and 24 h) were compared, patients in Group B had significantly lower VAS scores (*p* < 0.05). When the application of rescue analgesia was evaluated, there was a substantial difference between the groups (*p* < 0.001). Upon detailed evaluation of rescue analgesia, in the subgroup analysis where it was found to be significant, 51.5% of patients in Group B did not receive any rescue analgesia. In contrast, all patients in Group C received rescue analgesia (*p* < 0.05). Among the patients who received rescue analgesia twice, one patient (3%) was in Group B, whereas ten patients (30.3%) were in Group C (*p* < 0.05). When the change in VAS score over time across all time points was evaluated (time × group interaction), the interaction effect between time and group was significant (*p* < 0.001) (Table 3). When groups were evaluated for tramadol consumption, the values in Group C were greater (84 (74–156) vs. 0 (0–75) (median (25–75%)), and this difference was found to be significant (*p* < 0.001) (Table 3). When the QoR-15 score was assessed, the values in Group C were greater (129 [124–133]), whereas those in Group B were lower (122 [115–125] [median (25–75 percentiles]), and these differences were found to be significant (*p* < 0.001) (Table 3).

**Table 3:** VAS, analgesic data, and QoR-15 scores by group

Characteristics		Group C, *n*:33	Group B, *n*:33	*p* value
VAS (30. minute)		5(5–6)	3(3–4)	< 0,001
VAS (2. hour)		3(3–4)	2(1–2)	< 0,001
VAS(8. hour)		1(1–2)	1(1–1)	0,002
VAS(24. hour)		1(0–1)	0(0–1)	0,021
Rescue analgesia	No	0(0%)a	17(51,5%)b	< 0,001
	1	22(66,7%)a	15(45,5%)a	
	2	10(30,3%)a	1(3%)b	
	3	1(3%)a	0(0%)a	
Tramadol consumption (mg)		84(74–156)	0(0–75)	< 0,001
QoR-15		122(115–125)	129(124–133)	< 0,001

Continuous variables are expressed as medians (25–75 percentiles), while categorical variables are shown as n(%). Each superscript letter (a, b) indicates a subset of group categories that do not differ significantly from each other at the *p* = 0.05 level of statistical significance.

The distribution of rescue analgesia administration intervals for patients in the groups was as follows: between 30 min and 2 h, all patients in Group C received rescue analgesia; 48.5% of patients in Group B did; between 2 h and 8 h, 33.3% of patients in Group C and 3% of patients in Group B received rescue analgesia; and between 8 and 24 h, none of the patients in Group B received rescue analgesia, whereas 3% of patients in Group C did. When evaluated separately for the administration of rescue analgesia, the differences between the groups were statistically significant for the “30 minutes–2 hours” and “2–8 hours” intervals, whereas they were similar for the “8–24 hours” interval (*p* < 0.001, *p* = 0.001, and *p* = 0.314, respectively) (Table 4).

**Table 4:** Time-based rescue analgesic use by group

Time frame(h)	Group C, *n*:33	Group B, *n*:33	*p* value
0,5–2	33 (100%)	16(48,5%)	< 0,001
2–8	11(33,3%)	1(3%)	0,001
8–24	1(3%)	0(0%)	0,314

The chi-square test was applied. The data are presented as n(%).

## Discussion

Laparoscopic cholecystectomy has recently become the standard treatment. During laparoscopic surgery, intraperitoneal gas insufflation is necessary to facilitate surgical maneuvers and improve the quality of intra-abdominal visualization. In laparoscopic abdominal surgery, changes in respiratory mechanics are further exacerbated due to the impact of pneumoperitoneum on the diaphragm [22]. Protective lung ventilation strategies have been reported to be beneficial for reducing respiratory complications during the postoperative period [23, 24].

Upper abdominal surgery impacts respiration by reducing vital capacity; causing alveolar hypoventilation and hypoxemia; and decreasing inspiratory volume [25]. These changes are often attributed to reflex inhibition of the diaphragm. Recent studies have indicated that lung function and arterial gas levels are less affected following laparoscopic surgery than following open procedures [26, 27, 28]. A smaller decline in lung function may correspond to a reduced incidence of postoperative pulmonary complications. Postoperative analgesia techniques provide respiratory support by modulating pain pathways and enhancing the patient’s respiratory drive.

Given that the calculated mechanical power using ventilator parameters is instructive in promoting lung protection [29], our study’s results support the hypothesis that RSB and OSTAP block reduce the mechanical power for postoperative analgesia. Specifically, we found that the group receiving the block exhibited a lower mechanical power than the other groups, corroborating our hypothesis. Moreover, in Group B, a significant decrease was observed when comparing baseline mechanical power measurements with those taken before and after the administration of bridion. In contrast, an increase was noted in Group C. Applying these blocks for analgesia significantly reduces intraoperative mechanical power measurements before the operation begins.

Studies conducted to date have evaluated factors affecting respiration separately [30]. However, mechanical power, defined as the energy generated by mechanical ventilation and released into the respiratory system, has been proposed to be a critical determinant of the pathogenesis of VILI [14, 16, 31]. According to the classical equation of motion for the respiratory system, the energy applied to the lungs over time depends on the mechanical properties (elasticity and resistance), the applied tidal volume, the inspiratory flow, and the level of PEEP [32]. For example, reducing tidal volume and increasing the respiratory rate can either increase or decrease the total energy delivered to the lungs [16]. Experimental data, such as lung CT scan characteristics, suggest that mechanical power exceeding 12 joules/minute can lead to VILI, regardless of the variations in these component combinations [14]. Therefore, mechanical power should be considered a superior metric for modulating the final effect on VILI across different respiratory device settings [31].

Higher mechanical power during intraoperative mechanical ventilation has been shown to be significantly associated with an increased risk of postoperative respiratory failure requiring reintubation [21]. Additionally, in patients with ARDS, greater mechanical power has been linked to fewer ventilator-free days, longer stays in the intensive care unit, and greater in-hospital mortality [29]. These associations have been confirmed through secondary analyses of prospective data from patients enrolled in eight randomized controlled trials [33] and through another study of pooled data from six randomized controlled trials [34].

In contrast to previous studies, we focused on intraoperative changes in mechanical power during laparoscopic cholecystectomies. Our findings indicate that applying blocks for postoperative analgesia significantly reduces mechanical power. Considering the findings of other studies, monitoring mechanical power in patients with respiratory issues and employing blocks for postoperative analgesia appears promising. Furthermore, while mechanical power has primarily been assessed in intensive care unit patients, as shown in previous research [29, 35], intraoperative studies are lacking. Given the potential benefits of intraoperative mechanical power assessment, we chose to evaluate patients who underwent laparoscopic cholecystectomy.

In another study conducted by Santer P. and colleagues [21], every 5 J/minute increase in mechanical power led to a 1.31-fold greater probability of postoperative reintubation. The average mechanical power in this study was calculated at 7 J/minute, while it typically ranges between 20 and 30 J/minute in intensive care patients. In contrast, our study revealed a mechanical power of 6.08 ± 0.98 J/minute in Group C and 5.71 ± 0.88 J/minute in Group B. Furthermore, we noted a significant decrease in mechanical power in Group B after bridion administration [prebridion: -0.29 (-1.95 to 0.61) and postbridion: -1.00 (-2.02 to -0.18)], whereas an increase was observed in Group C [prebridion: 0.40 (-0.42 to 2.29) and postbridion: 1.68 (0.16 to 4.75)].

Combining parameters such as tidal volume and respiratory rate into a single value can assist clinicians in evaluating mechanical ventilation more comprehensively and determining the condition of patients’ lungs. This integrated approach helps move beyond reliance on a single ventilator parameter. High mechanical power values indicate that patients are at risk of postoperative reintubation, a critical hospital quality metric associated with increased mortality and hospitalization rates [36, 37, 38, 39]. Furthermore, an intraoperative increase in mechanical power of at least 2 J/minute is significantly associated with increased driving pressure and respiratory rate, leading to a 28% greater likelihood of developing postoperative respiratory failure [21].

After laparoscopic cholecystectomy, the predominant component of total postoperative abdominal pain, accounting for 50–70%, originates from the incision site. Pain from the pneumoperitoneum follows, contributing to 20–30% of cases, with pain directly from the cholecystectomy itself accounting for 10–20% of cases [40]. The multifactorial mechanisms triggering postoperative pain make its control challenging after LC.

Our study combined OSTAP and RSB to achieve superior analgesia, with both blocks applied bilaterally to cover a broader area. Consistent with many existing studies, our results revealed significantly lower VAS scores at postoperative assessments conducted at 30 min, 2 h, and 8 h. However, the VAS score did not significantly differ between the two groups at 24 h. Although a significant difference in analgesic consumption was observed between the groups in the first 8 h, which was correlated with the VAS score, no statistically significant difference was noted in consumption between 8 and 24 h.

Hebbard and colleagues described ultrasound-guided continuous OSTAP block, a variation of the TAP block that provides reliable unilateral supraumbilical analgesia [6]. Shin H and colleagues reported that OSTAP block offered better analgesia than conventional TAP block did in the first 24 h after surgery for patients who underwent LC [8]. Previous studies have reported that ultrasound-guided OSTAP blocks significantly reduce postoperative pain scores and opioid consumption in the first 24 h after surgery [8, 9, 41].

Ramkiran and colleagues [42] assessed the effectiveness of combining an RSB with an OSTAP block versus using an OSTAP block alone or traditional port site infiltration for alleviating postoperative pain after laparoscopic cholecystectomy. The authors reported significantly lower pain scores in the group that received the combination of blocks during the second postoperative hour. Despite these successful outcomes, some patients receiving OSTAP block may experience a patchy sensory block pattern in the lateral and posterior abdominal walls, resulting in discomfort after LC.

The RSB provides analgesia through midline incisions and laparoscopic procedures [43]. In particular, ultrasound-guided RSBs significantly reduce postoperative pain in single-incision laparoscopic cholecystectomy surgeries. While classic TAP blocks have been replaced mainly by OSTAP blocks in laparoscopic cholecystectomy, the issue of midline pain has not been sufficiently addressed [42, 44]. Although RSBs are specifically administered for midline pain, their combined use with other blocks in laparoscopic cholecystectomy has yet to be widespread.

Additionally, a study by Breazu CM and colleagues revealed that a preincisional OSTAP block effectively reduced intraoperative opioid consumption and opioid use over 24 h. Their study also reported significant decreases in VAS scores at 0, 2, 4, 6, 12, and 24 h postsurgery [9].

Our study also utilized the QoR-15, a validated measure frequently used across various clinical settings, to assess the comfort and quality of recovery after general anesthesia. This scale emphasizes postsurgical patients’ subjective well-being and satisfaction, covering their physical, mental, and emotional states. Higher QoR-15 scores indicate a better quality of recovery [45]. Opioid side effects can prolong hospital stays, increase treatment costs, delay the return to daily activities, and diminish the quality of recovery in postoperative patients. Effective control of postoperative pain not only reduces the risk of developing chronic pain but also decreases opioid consumption, accelerates recovery, alleviates anxiety, and shortens hospital stays [46, 47]. In line with these findings, our study revealed significantly higher QoR-15 scores at the 24-hour postoperative assessment in the group in which the block was applied. Similarly, Conghui Haove and colleagues reported that pain scores on the QoR-15 survey were lower in Group OA (opioid-based anesthesia), where opioids were used, than in Group OFA (opioid-free anesthesia), where opioids were not used, on postoperative days 1 and 2 [48].

### Limitations

This study has several limitations. First, the mechanical power was only calculated three times during the surgical procedure rather than being continuously monitored. This infrequent measurement may not accurately reflect the dynamic changes in mechanical power applied to patients throughout surgery. Second, a computer could have performed our measurements automatically, which could lead to potential inaccuracies. Third, we could only partially control for factors affecting airway pressure, such as increased secretions.

## Conclusions

Our study revealed positive outcomes related to respiratory mechanics through the use of OSTAP and RSB blocks, specifically regarding reduced mechanical power. While these findings warrant confirmation in future studies, the intraoperative assessment of mechanical power, if facilitated by real-time calculations through computer simulations, could aid in identifying patients at risk of postoperative respiratory failure.

## Electronic supplementary material

Below is the link to the electronic supplementary material.

Supplementary Material 1

## Data Availability

To access the raw data analyzed in this study, please get in touch with the corresponding author, Esma Karaarslan, MD, via email at esmaayvaz@gmail.com or by phone at +90 505 731 70 61. I can share the data upon request if you want to review the results; however, it is inaccessible via a link.
