# Supplementary material for: The effect of bilateral rectus sheath and oblique subcostal transversus abdominis plane blocks on mechanical power in patients undergoing laparoscopic cholecystectomy surgery: a randomized controlled trial
Source: BMC Anesthesiol. 2025 Apr 16;25:186. doi: 10.1186/s12871-025-03062-6 (PMC12004598; doi:10.1186/s12871-025-03062-6)
Supplement: Supplementary file 1 — Supplementary Material 1 [file 12871_2025_3062_MOESM1_ESM.docx]

## Enrollment

Randomized (n=69 )

Excluded (n= 7)

♦  Not meeting inclusion criteria (n=4)

♦  Declined to participate (n= 3)

Assessed for eligibility (n=76)

Analysed (n= 33)
♦ Excluded from analysis (n=0)

Analysed (n= 33)
♦ Excluded from analysis (n= 0)

Discontinued intervention (n= 1)

• Protocol breach (n=0)

 • Lost to follow-up (n=1)

  Discontinued intervention (n=2)

 • Protocol breach (Converted to open surgery n=1)

• Lost to follow-up (n=1)

Allocated to group C (n=35 )

♦ Received only intravenous analgesia protocol (n=35)

♦ Did not receive the allocated intervention (n= 0 )

Allocated to group B (n=34)

♦ Received RSB and OSTAP block with local anesthetic (n=34 )

♦ Did not receive the allocated intervention(n=0)

## Allocation

## Follow-Up

## Analysis

Figure1. Flow diagram of thestudy. (OSTAP: oblique subcostal transversus abdominis plane, RSB: rectus sheath block)
